# Study of Phospholipase A2 Levels and Its Comparison With Procalcitonin Levels in Patients With Sepsis Admitted in a Tertiary Care Hospital, Karnataka, India

**DOI:** 10.7759/cureus.50890

**Published:** 2023-12-21

**Authors:** Suma D Borra, Dnyanesh N Morkar

**Affiliations:** 1 Internal Medicine, Jawaharlal Nehru Medical College and Hospital, Belagavi, IND

**Keywords:** comparison, biomarker, procalcitonin, phospholipase a2, sepsis

## Abstract

Introduction: Sepsis is a complicated host response to infection involving organ failure which ultimately causes death of the host. Procalcitonin (PCT) is an effective marker used to diagnose sepsis but until now, there has been no ideal marker for sepsis. Phospholipase A2 (PLA2) also increases infections; however, only a few studies have assessed its capacity as a biomarker to diagnose sepsis. Thus, we aimed to examine PLA2 and compare its diagnostic capacity and accuracy with PCT as a biomarker of sepsis.

Material and Methods: Our study was a hospital-oriented cross-sectional study. Our study group included 80 patients of both sexes older than 18 years, meeting the quick sequential organ failure assessment (qSOFA) or systemic inflammatory response syndrome (SIRS) criteria of ≥2, hospitalized in a tertiary care hospital in Karnataka, India from January 2021 to December 2021. Out of them, 59 were found to have sepsis. Samples of all the patients were evaluated for relevant parameters, and data were statistically analyzed using SPSS v21 running on Windows 10. The statistical significance was set at p-value <0.05.

Results: The mean PCT and PLA2 were significantly raised in sepsis patients compared to non-sepsis patients. Out of 59 septic patients, 45.76% had positive blood cultures, and 16.95% had positive urine culture reports. In blood cultures, the most common Gram-positive organism found was *Staphylococcus*, and the most common Gram-negative organism was *Enterobacter*. In urine cultures, *Escherichia coli* was the most common species. PLA2 was significantly higher in patients with bacterial etiology and Gram-positive cultures. The diagnostic capability, sensitivity, specificity, and accuracy of PLA2 were demonstrably higher than those of PCT.

Conclusion: Our study proves that PLA2 is a much better and more efficient biomarker in sepsis than PCT. The diagnostic capacity and accuracy of PLA2 clearly surpass PCT, so using PLA2 in sepsis as a biomarker can help clinicians in deciding on timely and appropriate management to speed the recovery of patients.

## Introduction

Sepsis is a medical condition that is a complication of infection, affecting many organs of the host and can ultimately lead to death [1]. It is now considered a global health concern, as a recent survey observed that 11 million sepsis patients died out of 48.9 million cases worldwide, which equated to 19.7 % of overall deaths in 2017 [2]. In the past few decades, the occurrence and mortality rate of sepsis have grown significantly. Sepsis has a multifaceted pathophysiology and the heterogeneity of this syndrome makes timely and reliable diagnosis of the disease difficult, ultimately leading to a lack of time-specific management [3-5].

The traditional diagnostic criteria for sepsis in suspected infection cases is a sequential organ failure assessment (SOFA) or systemic inflammatory response syndrome (SIRS) score ≥2 [6]. However given the many limitations of these criteria, a simplified method namely quick SOFA (qSOFA) came into use. Though qSOFA is extremely specific, Williams et al. found it to be not sensitive enough, making it unsuitable for early detection of sepsis [7]. The second most important tool for sepsis diagnosis is blood cultures, but the procedure takes time and may show false-negative results mainly after antibiotic use [8]. As a result, clinicians cannot rely only on blood culture for early diagnosis of sepsis.

If sepsis is appropriately detected and managed within 1 hour of infection, the survival rate will be above 80% but if sepsis is diagnosed and managed after 6 hours of infection, then the survival rate falls to 30% [9]. So, it is essential to trace a biomarker for early detection of the disease. Despite the wide-ranging exploration of numerous biomarkers, an adequately sensitive and specific ideal biomarker has not yet been identified.

More than 250 sepsis biomarkers have been explored as emerging biomarkers to evaluate their diagnostic or prognostic value such as presepsin, D-dimer, C-reactive protein, interleukin-6, pancreatic stone protein, bactericidal permeability-increasing protein, brain natriuretic peptide, group II phospholipase A2 (PLA2), procalcitonin (PCT), and more [10]. Numerous studies have proven PCT to be an efficient and promising marker for SIRS over other markers [10,11]. Out of the list of markers, phospholipase A2 (PLA2) is the least explored marker for sepsis. Numerous small-scale clinical trials have already revealed a positive correlation between PLA2 and successful diagnosis of bacteremia making it a valuable biomarker [12,13]. Thus, in our study, we have compared the efficacy as a sepsis marker of PLA2 with that of PCT.

## Materials and methods

Our research was hospital-oriented and employed a cross-sectional study design. We enrolled 80 patients in the study, who fulfilled the inclusion criteria and were hospitalized in a tertiary care hospital in Karnataka, India from January 2021 to December 2021. Informed consent was taken from all the patients or relatives after obtaining ethical clearance from the Institutional Ethics Committee (MDC/DOME/17 dated 25-01-2021). Patients of both genders who were 18 years old or older and who met the qSOFA or SIRS criteria of ≥2, along with clinical examination were included in the study. To meet the qSOFA criteria, a patient needs a systolic blood pressure ≤ 100 mmHg, a respiratory rate ≥ 22/min, or a change of consciousness. If at least two scores are present, this can be considered a high-risk sepsis population [7]. SIRS is manifested by at least two of the following criteria: temperature < 36°C or >38°C, respiratory rate ≥ 20/min, heart rate >90 beats/min, and white blood cell count <4,000/mm^3^ or >12,000/mm^3^ [6]. Further, the serum samples of all the participants were sent to the laboratory for analysis within 24 hours of their admission to the hospital, and a complete evaluation along with PLA2 and PCT was done. A culture examination was also conducted on the serum and urine of the patients. Based on the PCT level and culture report, 59 patients were found to have sepsis and 21 patients were sepsis-free. The patients on antibiotics for >3 days and the patients with a history of rheumatoid arthritis, asthma, systemic lupus erythematosus, and malignancies were excluded from the study.

Statistical analysis

The assessed data were tabulated on Microsoft Excel version 16.48 after the generation of the proper template. Data were statistically analyzed using SPSS v21 running on Windows 10. Descriptive and categorical variables were analyzed. The strength association (p-value) was calculated using an unpaired t-test or the Mann- Whitney U test (non-parametric). To assess the threshold of biomarkers, the receiver operating characteristic (ROC) curve was used, and the cut-off values were calculated using the Youden index formula. All the tests were two-tailed tests, and the statistical significance was set at a p-value <0.05.

## Results

**Figure 1:**
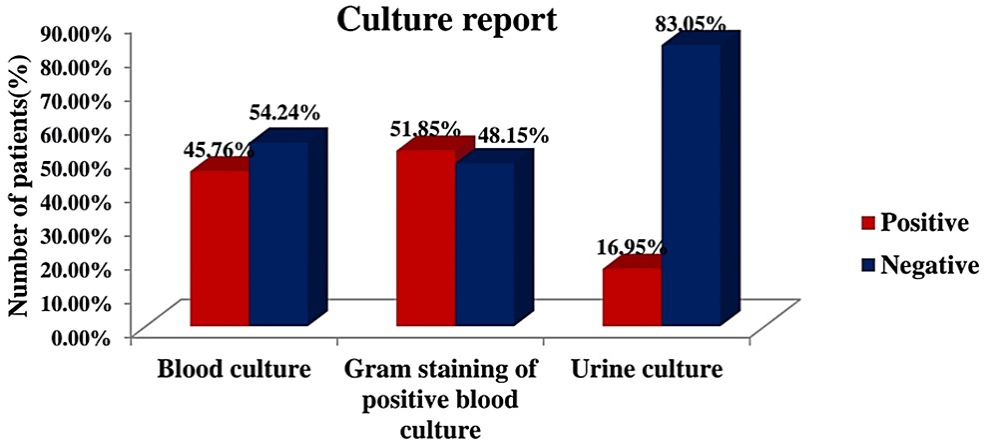
Distribution of Sepsis Patients Based on Culture Report

The study was composed of 80 patients, with the mean age of sepsis patients as 55.60±17.20 years and the mean age of non-sepsis patients as 62.40±12.97 years. The difference in mean age was non-significant. Our study had 34 males (57.63%) and 25 females (42.37%) in the sepsis group, and the non-sepsis group also contained more males than females (males: 61.90%; females: 38.09%). Gender had no association with sepsis. Table 1 shows that the mean PCT and PLA2 levels in the sepsis patients were observed to be 41.95±39.58 and 105.20±60.06 ng/dl respectively. Both biomarkers were significantly low in non-sepsis patients (0.34±0.71 and 17.90±4.90 ng/dl, respectively). Figure 1 illustrates the blood and urine culture examination of the sepsis patients. Out of 59 patients, 27 (45.76%) were found to have positive blood culture reports, and 32 (54.24%) had negative reports. We conducted further Gram staining of the positive samples and found that 14 (51.85%) were Gram-positive and 13 (48.15%) were Gram-negative. The most common Gram-positive organism in blood culture was *Staphylococcus*, and the most common Gram-negative organism was *Enterobacter*. In the sepsis patients, the urine culture report was found to be positive in 10 (16.95%) patients, with *Escherichia coli* (*E. coli*) being the most common causative organism.

**Table 1:** Variables of Sepsis Patients

Parameter		Sepsis Patients	Non-sepsis Patients	p-value
Mean age (years)		55.60±17.20	62.40±12.97	0.102
Sex n(%)	Males	34 (57.63%)	13 (61.90%)	0.800
	Females	25 (42.37%)	8 (38.09%)	
PCT (ng/dl)		41.95±39.58	0.34±0.71	0.001
PLA2 (ng/dl)		105.20±60.06	17.90±4.90	0.001

Table 2 depicts the variation of mean PCT and PLA2 levels based on blood culture reports. Mean PCT was non-significantly elevated in blood culture-positive patients (46.08±41.14 ng/dl) when compared to blood culture-negative patients (37.88±38.14 ng/dl). However, mean PLA2 was observed to be significantly raised in blood culture-positive patients (143.44±60.65 ng/dl) compared to blood culture-negative patients (72.85±36.21 ng/dl).

**Table 2:** Variation of PCT and PLA2 Based on Blood Culture Reports PCT: procalcitonin; PLA2: phospholipase A2

Parameter	Blood Culture Positive (Mean±SD)	Blood Culture Negative (Mean±SD)	p-value
PCT (ng/ml)	46.08±41.14	37.88±38.14	0.431
PLA2(ng/ml)	143.44±60.65	72.85±36.21	<0.001

Further, sepsis patients were categorized into two groups based on whether the etiology of the infection was bacterial or viral. Out of total sepsis patients, 40 patients were found to have bacterial etiology and 19 were found to have viral etiology. As shown in Table 3, mean PCT and PLA2 were both found to be significantly higher in patients with bacterial etiology (46.11±39.14 and 113.84±57.49 ng/dl, respectively) than in patients with viral etiology (2.12±3.01 and 28.50±2.44 ng/dl, respectively).

**Table 3:** Variation of PCT and PLA2 Based on Etiology PCT: procalcitonin; PLA2: phospholipase A2

Parameter	Bacterial Etiology (Mean±SD)	Viral Etiology (Mean±SD)	p-value
PCT (ng/ml)	46.11±39.14	2.12±3.01	0.008
PLA2(ng/ml)	113.84±57.49	28.50±2.44	<0.001

As shown in Table 4, when we conducted Gram staining of the positive blood cultures, we observed that the mean PCT was lower in Gram-positive patients (43.20±41.48 ng/dl) than in Gram-negative patients (51.46±42.22 ng/dl); this difference in mean value was not significant. As far as mean PLA2 is concerned, it was significantly higher in Gram-positive patients (176.57±45.73 ng/dl) than in Gram-negative patients (110.75±56.18 ng/dl).

**Table 4:** Variation of PCT and PLA2 Based on Gram staining PCT: procalcitonin; PLA2: phospholipase A2

Parameter	Gram-Positive (Mean±SD)	Gram-Negative (Mean±SD)	p-value
PCT (ng/ml)	43.20±41.48	51.46±42.22	0.713
PLA2(ng/ml)	176.57±45.73	110.75±56.18	0.004

**Figure 2:**
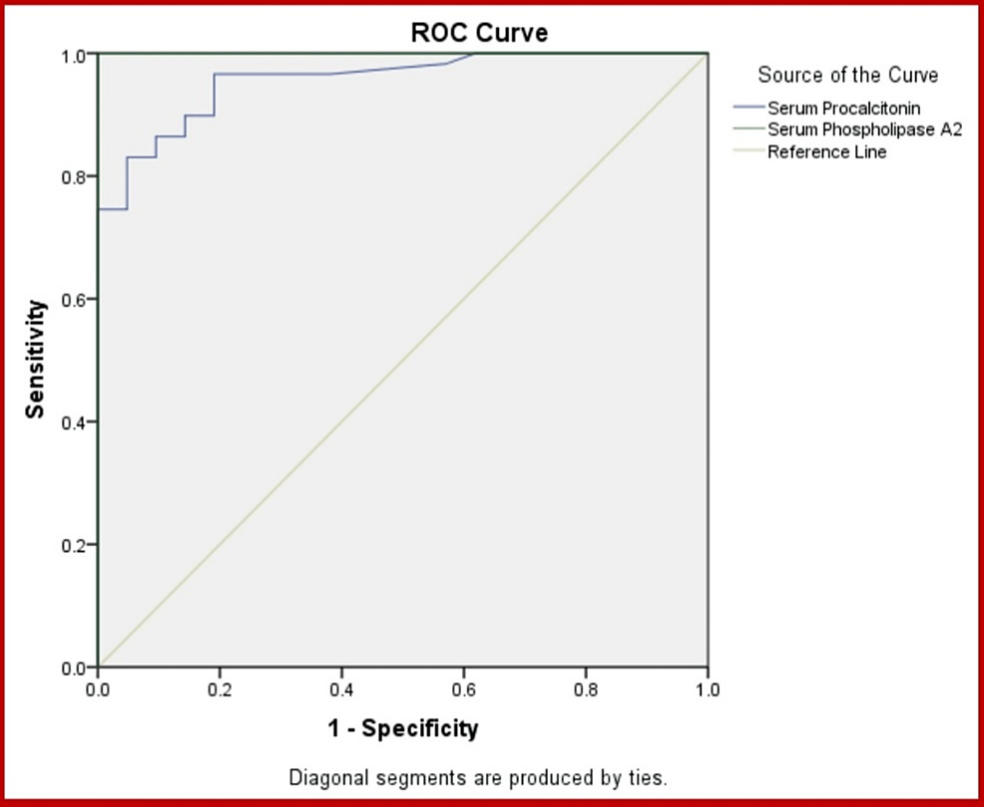
ROC Curve Representing Sensitivity and Specificity of PCT and PLA2 ROC: receiver operating characteristic; PCT: procalcitonin; PLA2: phospholipase A2

Table 5 and Figure 2 depict the comparison of diagnostic capacity and accuracy of PLA2 and PCT as biomarkers of sepsis. On the assessment of the area under the curve (AUC), PLA2 was found to have a significantly higher AUC of 1.00 compared to PCT with an AUC of 0.957. Further, both the specificity and the sensitivity of PLA2 (90.1% and 98.6%, respectively) were assessed to be higher than those of PCT (88.9% and 95.5%, respectively). We observed a higher positive predictive value (PPV) of 89.4% and negative predictive value (NPV) of 92.3% of PLA2 when compared to PCT, which had a PPV of 82.6% and an NPV of 88.1%. As far as the accuracy of both biomarkers is concerned, it was also observed to be more in the case of PLA2 (99.0 %) than PCT (95.7 %).

**Table 5:** Diagnostic Capacity and Accuracy of Each Biomarker to Detect Sepsis

Test Result Variable		PCT	PLA2
Cut-off value		0.820	24.500
Area		0.957	1.00
Asymptotic Sig.		0.000	0.001
Asymptotic 95% Confidence Interval	Lower Bound	0.916	1.000
	Upper Bound	0.997	1.000
Sensitivity (%)		95.5	98.6
Specificity (%)		88.9	90.1
PPV (%)		82.6	89.4
NPV (%)		88.1	92.3
Accuracy (%)		95.7	99.0

## Discussion

The present study was conducted on 80 patients hospitalized in a tertiary care hospital in Karnataka and considered the qSOFA score. The study was conducted to compare the efficacy of PLA2 and PCT as biomarkers of sepsis. The mean ages of the sepsis and non-sepsis patients in the current study were comparable. The mean age of the sepsis patients in our study was 55.60±17.20 years, and there were more males (57.63%) than females (42.37%) in this group. This finding is in agreement with studies by Tan et al., Deme et al., and Anglès et al. as well as other previous studies, as they also found similar mean ages between the groups and a male preponderance [2,14-17]. Another study by Tan et al., also documented a similar mean age, although the distribution of males and females was equal in their study [18].

The current study observed significantly higher mean PCT and PLA2 in sepsis patients when compared to the non-sepsis group. This was supported by Tan et al. and Anglès et al. who also found these biomarkers to be elevated in sepsis patients [14,16]. Tambo et al. and Varela-Patiño et al. also found a strong association between PCT and sepsis [19,20]. The reason behind raised PCT in sepsis is well documented and self-explanatory, as PCT behaves as an acute phase reactant, increasing during inflammation and sepsis. In the acute phase of inflammatory response during sepsis, PLA2 enzymes are mostly associated with high-density lipoproteins (HDL), which are the major source of phospholipids in plasma. These secretory PLA2-modified HDL have potent anti-inflammatory characteristics.

Further, in the blood culture examination of our study, 45.76% of those tested had positive blood culture reports, and 54.24% had negative reports. This finding disagrees with those of Huang et al. and Deme et al., who documented only 29.8% and 19.6% positive reports, respectively [15,21]. Gram staining of positive blood culture reports in the current study showed that 51.85% were Gram-positive and 48.15% were Gram-negative. This finding is also in contrast to the study by Deme et al., who observed a much lesser proportion of Gram-positive organisms (21.7%) [15]. In the present study, the proportion of sepsis patients having positive urine culture was found to be 16.95%, which does not align with the study by Tan et al., who found only 9.8% positive urine reports among sepsis patients [18]. Another study by Deme et al. found much higher positive urine culture reports (79.5%) in sepsis patients [15]. Our study found the most common Gram-positive organism in blood cultures to be *Staphylococcus* and the most common Gram-negative organism to be *Enterobacter*. In urine cultures, *E. coli* was the most common species. This outcome is supported by Deme et al., who also isolated *E. coli* in urine, though they found *Acinetobacter baumannii* to be more common in the blood culture [15]. This outcome aids the clinicians in starting prompt therapy to prevent the patients from developing antimicrobial resistance in the long run.

In this study, both, PCT and PLA2 were significantly higher in patients with bacterial etiology than those with viral etiology. This finding conforms with those of Tan and Goh and Rintala et al., who also indicated that PLA2 was related to the severity of sepsis and bacterial infections [12,22]. Nandu et al. also demonstrated a significant elevation of PLA2 in culture-positive patients [23]. However, PCT was found to be elevated non-significantly. So, the level of PLA2 can act as a guiding principle in introducing antibiotic therapy to only the relevant group and avoiding pointless antibiotic use in other groups.

Further, our study compared the diagnostic capacity and accuracy of PCT and PLA2 as biomarkers of sepsis. PLA2 was found to have a significantly higher AUC (1.00) compared to that of PCT (0.957). This finding is strongly supported by Anglès et al., who also observed a significantly higher AUC of PLA2 (0.896) than PCT (0.765) [16]. The current study documented PLA2 as an excellent screening biomarker of sepsis with greater sensitivity and specificity than PCT. This outcome aligns with the findings of Tan et al., who also found high sensitivity and specificity of PLA2 (94%) [18]. Another study by Muzlovic et al. observed the specificity of PCT as 100%, but sensitivity was only 82% [24]. Studies by Godnic et al., Bauer et al., and Spoto et al. also found much less sensitivity and specificity of PCT than the current study [25-27]. Our study observed higher PPV (89.4%) and NPV (92.3%) of PLA2 when compared to the PPV (82.6%) and NPV (88.1%) of PCT. This finding is in concordance with Tan et al., who also found higher PPV and NPV of PLA2 (97% and 90%, respectively) [18]. However, Mearelli et al. observed equal NPV of PCT and PLA2 (93%) [28]. Another study by Deme et al. found the PPV of PCT to be 95.0%, but NPV was only 38.5% [15].

As far as the accuracy of both biomarkers is concerned, the accuracy of PLA2 (99.0%) was calculated to be higher than that of PCT (95.7%). This finding is also strongly supported by Tan et al., who calculated the higher accuracy of PLA2 [18]. Venugopalan et al. observed even lower sensitivity, specificity, NPV, PPV, and accuracy of PCT than our study [29]. When Tan et al. compared PLA2, PCT, and other markers, they also found the AUC, sensitivity, specificity, NPV, PPV, and accuracy of PLA2 to be higher than those of PCT in bacterial infection patients, which strongly supports our study [14]. The data of our single-centric study strongly suggested PLA2 was a better marker in sepsis than PCT, which is in agreement with Anglès et al. who also proved PLA2 to be a superior marker than PCT in septic shock [16]. Berg et al. also stated that secretory PLA2 has the potential to be a marker in early diagnosis of patients presenting with possible sepsis [30]. Tan et al. also found that PLA2 had the most significant ROC, followed by other markers and PCT [14]. The same order was observed in the specificity and sensitivity of the components tested. The use of PLA2 will thus help in early diagnosis and the reduction of patient morbidity and mortality.

In light of these findings, it is important to note that the host immune response to sepsis and bacterial infection involves a series of complex inflammatory pathways, and it is unlikely that any individual biomarker could be used to accurately classify and predict the outcomes of these conditions. Thus, using a combination of biomarkers may have the theoretical advantage of improving both, the sensitivity and the specificity of the tested outcomes, leading to greater diagnostic accuracy.

The limitation of our study was that being a single-centric hospital-based study, all patients were selected solely from a tertiary care center in Karnataka, making it prone to selection bias. The small sample size was the other limitation.

## Conclusions

The study was conducted to compare the diagnostic values of PLA2 and PCT as biomarkers in sepsis. Our study proves that PLA2 is a much better and more efficient biomarker for sepsis than PCT. The diagnostic capacity and accuracy of PLA2 are clearly superior, so applying PLA2 as a sepsis biomarker can potentially assist clinicians in deciding the best way to administer antimicrobial therapy to expedite the recovery of patients. Thus, it can help ensure patients receive intensive management, resulting in decreased mortality rates. However, given the complexities of sepsis, finding an ideal biomarker is improbable, so a panel of biomarkers including PLA2 could be most beneficial. Further, a multi-centric study involving a larger sample size is recommended.
